# Plant genome modification: from induced mutagenesis to genome editing

**DOI:** 10.18699/VJGB-22-83

**Published:** 2022-11

**Authors:** А.B. Shcherban

**Affiliations:** Institute of Cytology and Genetics of the Siberian Branch of the Russian Academy of Sciences, Novosibirsk, Russia Kurchatov Genomic Center of ICG SB RAS, Novosibirsk, Russia

**Keywords:** induced mutagenesis, transgenesis, genome editing, nucleases, CRISPR/Cas9, pathogen, resistance, yield, индуцированный мутагенез, трансгенез, геномное редактирование, нуклеазы, CRISPR/Cas9, патоген, устойчивость, урожайность

## Abstract

The snowballing growth of scientif ic data obtained using modern techniques of genome editing (GE) calls for their critical evaluation and comparison against previously applied methods such as induced mutagenesis, which was a leading method of genome modif ication for many decades of the past century, and its application has resulted in a huge diversity of cultivars. However, this method was relatively long and included a number of stages from inducing multiple mutations using different mutagenic factors to crossing and selecting the most valuable cultivars for several generations. A new technology of genetic engineering and transgenesis enabled us to radically reduce the time required to obtain a new genetically-modif ied cultivar to one generation and make the modif ication process more effective and targeted. The main drawback of this approach was that an introduced transgene might uncontrollably affect the other genes of a recipient plant, which led to the limitations imposed on transgenesis application in many countries. These limitations have been effectively surmounted thanks to the development of GE techniques allowing for a precise modif ication within a single gene that in many characteristics make it similar to a natural allele (especially when it comes to ribonucleoprotein complexes), which has paved the way for wide application of GE in routine breeding. The paper reviews the main stages of GE development in its application in plants. It provides short descriptions of different GE techniques, including those using protein editors such as zinc-f inger and transcription activator-like effector nucleases (TALEN), and the CRISPR/Cas9 technology. It lists a number of achievements in using GE to produce new cultivars of higher yield that are resistant to unfavorable factors and have good nutritional properties. The review also considers the de novo domestication approach, which allows for faster obtaining of new cultivars from natural varieties. In the conclusion, the future ways of GE development are discussed.

## Abstract (ru)

Лавинообразный рост научных данных, полученных с помощью современных методов геномного редактирования (ГР), обуславливает актуальность их критического осмысления и сопоставления с предыдущими методами модификации генома. В обзоре дана характеристика основных этапов развития методов модификации генома применительно к растительным объектам. Технология индуцированного мутагенеза лидировала в течение многих десятилетий прошлого века, с ее помощью получено огромное разнообразие сортов культурных растений. Однако этот процесс был довольно длительным и включал целый ряд стадий: от индукции множественных мутаций с помощью мутагенных факторов до этапов скрещивания и отбора наиболее ценных форм на протяжении ряда поколений. Пришедшая на смену технология генной инженерии (трансгенеза) позволила радикально сократить время получения новых генетически модифицированных форм до одного поколения, сделать процесс модификации более эффективным и целенаправленным. Но наряду с этим она имела главным недостатком возможность неконтролируемого влияния вводимого трансгена на другие гены растения-реципиента, что привело к существенным ограничениям применения трансгенеза во многих странах. Эти ограничения в настоящее время успешно преодолеваются с развитием методов ГР, позволяющих очень точно, в пределах одного гена, осуществлять модификацию, которая по своим свойствам практически не отличается от природного аллеля гена (особенно в случае использования рибонуклеопротеиновых комплексов), что дает возможность избежать ограничений на применение этой технологии в практической селекции. Приведена краткая характеристика различных методов ГР, включая использование белковых редакторов, ZF- и TALEN-нуклеаз, а также наиболее перспективный метод – CRISPR/Cas9. Перечислен ряд научных результатов по созданию с помощью этих методов новых форм растений: устойчивых к неблагоприятным факторам, с повышенной урожайностью и ценными питательными свойствами. В рамках обзора рассматривается новый подход «доместикация de novo» с целью ускоренного получения культурных растений из природных форм. Обсуждаются дальнейшие пути развития методологии ГР.

## Introduction

Continuous accumulation of spontaneous mutations is the foundation of evolution in living organisms. Mutation frequency depends on the features of a creature’s genetic apparatus and varies from 10–9 to 10–12 nucleotides/cell generations. Mutations commonly occur due to disrupted key biological processes such as DNA replication, reparation and recombination (Jonczyk et al., 1988; Banerjee et al., 1990), and only their insignificant part becomes involved in the evolutionary process while others are eliminated during selection. The mutations induced by chemical agents, radiation and other factors are random but of high frequency that provokes a huge number of mutation events in a genome (Sakuraba et al., 2005). However, selecting useful alleles and their combinations is a long-term process that involves crossing with wild genotypes and cultivating necessary ones for several generations. Nevertheless, significant number of modern cultivars have resulted from the breeding programs using induced mutagenesis that were launched in the beginning and middle of the 20th century, in other words, they are partially a subproduct of nuclear technology development

The second method for obtaining new versions of genes lies with genetic engineering and transgenesis. The main advantage of this approach, if compared to induced mutagenesis, is that is allows for fast and dedicated effect on a certain trait through an induced alien transgene, which significantly reduces the time required to obtain a genetically modified organism (GMO) (Khush, 2012). However, along with the advantages, the method has certain drawbacks that will be discussed in a separate section below

The further advancement of genome modification technologies is related to improved dedicated delivery of vector molecules so they could directly affect certain genetic loci, which has been implemented in the gene targeting strategy (Hall et al., 2009). The strategy allows one to overcome the main disadvantage of transgenesis that is a possibility for a transgene to introgress into different genomic regions, makes the expected effect more targeted and prevents off-target editing of other genes. Its foundation was initially based on the phenomenon of homologous recombination between a vector’s DNA sequence and a genomic DNA sequence homologous to it (Smithies et al., 1985; Capecchi, 1989). The process results in either deletion of a gene or its part so the gene loses its functionality (gene knockout); or insertion of additional sequence; or modification of certain base pairs (point mutation). Genetic targeting is widely used in human and animals. In particular, it is applied to study the genetic diseases in cell lines for which a knockout or a modification of a potentially pathogenic gene can be performed in vitro (Sur et al., 2009). Together with homologous recombination, the genomes of eukaryotic organisms employ non-homologous end joining (NHEJ) that may generate unpredictable frequent mutations during DNA repair (Guirouilh-Barbat et al., 2004).

Another big advancement that has significantly increased the efficacy of genetic targeting has become the development of artificial endonucleases such as meganucleases, zinc-finger (ZF), transcription activator-like effector (TALEN) and Cas9 site-specific nucleases. It is the use of those nucleases that has given birth to a new specific term “genome editing (GE)” although today it refers to any methods of gene modification (Bak et al., 2018).

ZF and TALEN nucleases are used in combination with targeting proteins such as ZF domains and the proteins similar to TAL effectors, respectively. In case of Cas9 nucleases, it is CRISPR RNA that gave birth to the CRISPR/Cas9 technology that has revolutionized GE being the least laborious, relatively inexpensive and most precise and effective technology to the date. For the time passed since its introduction in 2012, it has been applied for editing of a huge number of living organisms from humans to yeast (Khlestkina, Shumny, 2016).

In what follows, the results obtained in plants with different genome modification techniques will be considered.

## Induced mutagenesis

The effect radiation has on heredity was first demonstrated by Russian botanist Georgy Nadson (Nadson, Philippov, 1925) and American genetic scientist Hermann J. Muller (Muller, 1927). Their discovery fostered multiple genetic studies that went in parallel with the development of wave and nuclear physics. Among such studies were those carried out by prominent Russian scientists including A. Sapegin who studied radiation-induced mutagenesis in common wheat (Sapegin, 1930), and N. Timofeev-Resovsky who started a new direction in radiation genetics (Timofeeff-Ressovsky, 1929). At the same time, chemical mutagenesis was studied by N. Koltsov and his disciple I. Rapoport whose achievements became crucial for applying the method in plant selection (Rapoport, 1946).

Since the 1930th, both radiation and chemical mutagenesis techniques have been used all over the world to produce more than 3200 cultivars of 200 species (https://mvd.iaea.org).

In this respect, Russia takes the fourth place (6.7 % of mutagenic cultivars) after China, India and Japan (Ahloowalia et al., 2004). In our country, the mutant plants have been used to obtain the cultivars of winter/spring wheat, barley, soybeans,lupin, oat, beans, etc. For instance, common wheat cultivar Novosibirskaya 67 was created using the radiation technique and became the fruit of the joint efforts of the breeders of Novosibirsk Experimental Station and Institute of Cytology and Genetics of Siberian Branch of the USSR Academy of Sciences (Cherny, 1982). For a long time, the cultivar had remained the leading crop of Western Siberia in terms of planted areas for it combined high productivity, excellent baking properties and was resistant to a number of diseases. The scientists of Research Institute of Oil Crops (Krasnodar) used chemical mutagenesis to produce Pervenets, a new sunflower cultivar whose oil quality was comparable to that of olive trees (Russian Solar Flower, 2007). 

The dwarfism mutation was used by N. Borlaug to breed the cultivars of non-lodging high-yielding common wheat that paved the way for the so-called green revolution in the middle of the last century (Gaud, 1968). E. Sears and F. Elliot used experimental mutagenesis in combination with long-term hybridization to transfer the loci of resistance to rust and smut from the wild varieties of goat and wheat grass to common wheat (Kilian et al., 2011). G. Stubbe (DRG) applied 5-time X-ray irradiation and selection in several generations of smallfruited wild tomato to increase its fruit to the size commonly observed in cultivated tomato (Stubbe, 1957).

## Transgenesis

The next technology to obtain new gene versions that came onto stage was genetic engineering or artificial transgenesis. In its essence the technology is introduction of an alien gene (transgene) into a living organism that facilitates the last to obtain predictable and inheritable traits. In plants, transgenes are delivered using the specialized vectors created using the tumor-inducing (Ti) plasmids of agrobacteria (Weising et al., 1988). Since all plant species have similar genetic code, it means a transgenic organism is able to express alien genes.

This approach had multiple advantages if compared to induced mutagenesis. First, it significantly widened the possibility for dedicated modification of living organisms because transgenes could have the traits untypical for a recipient, so they could not be obtained using mutagens (e. g., synthesis of pharmaceutical, insecticide and other agents in plants). Second, the technology significantly reduced the scale and duration of selection especially after such markers as antibiotic resistance and reporter genes were introduced into vector DNA and allowed for fast and effective identification of genetically modified organisms (GMO). In terms of fundamental science, transgenic organisms became a convenient model for studying the functions of a particular gene and their phenotypical manifestations

Genetic engineering has been used to obtain multiple genetically modified cultivars of corn, rice, soybean, cotton, rape, potato and others whose farming areas take hundreds of millions of hectares all over the world (Genetically Engineered Crops…, 2016). One of the examples of transgenic plants is Golden Rice that has high content of β-carotene, a precursor to vitamin A whose deficiency leads to xerophthalmia, a widespread eye condition in South-East Asia. To obtain this cultivar, a gene of phytoene synthase (Narcissus) was introduced into a local variety using the bioballistics technique (Burkhardt et al., 1997). Another example of successful transgenesis in agriculture is transgenic soybean.

Its cultivars are widely represented on the market and are known for their resistance to different herbicides such as Roundup (glyphosate), glufosinate, Dicamba. Others contain the gene of Bacillus thuringiensis (BT), whose toxin make them resistant to insects (https://www.isaaa.org/gmapproval database).

An analogous transgene was introduced to cotton and made it resistant to the cotton budworm, a common pest for this species (Wu et al., 2008). Genetic engineering has also produced the transgenic varieties of cotton, maize and rape resistant to herbicides (Tan et al., 2005; Karthik et al., 2020), and that of maize resistant to insects (Lundmark, 2007) and many others.

All these examples prove the technology has been successfully applied in the agricultural sector of such countries as the USA, China, India, Argentina, Canada and others where industrial agriculture and transgenic plants were permitted unlike the majority of countries where the using and growing of GMOs was prohibited or unlike Russia that only allowed for import of GMOs as food products, forage, and research objects (Dudin, 2020). Although, most of GMO-related concerns have been due to prejudices or the rivalry of agrochemical companies, it is still cannot be stated that all such concerns have been completely ungrounded. GMOs present a certain danger for ecosystems, e. g., if we have produced herbicideresistant plants, how can we be certain that these genes will not be transferred to weeds by pollen while cross hybridization (Schütte et al., 2017).

There is also a risk that a transgenic plant can affect nontarget organisms such as plants possessing BT-toxin genes can kill non-hazardous insects (Marvier et al., 2007). The long-term consequences of transgenesis remain unclear since a transgene can enter different regions of a genome and ruin other genes’ expression. As for their direct harm to human health, multiple scientific research has shown that GMOs and their products are of no more harm than traditional crops (König et al., 2004).

## Genome editing

The basis of GE is dedicated changing of a limited gene region that may be achieved in different ways. Considering the early days, the first experiments were applying oligonucleotides for DNA editing, e. g., two genes (defective green fluorescent protein and acetolactate synthase) of tobacco and corn were edited using chimeric RNA/DNA oligonucleotides in 1999 (Beetham et al., 1999; Zhu et al., 1999). In the last case, the editing resulted in a low-frequent resistance to imidazoline and sulfonylurea. This study was followed by analogous works to alter these and other species of plants (Zhu et al., 2000; Kochevenko, Willmitzer, 2003; Okuzaki, Toriyama, 2004), but the effectiveness of the techniques remained comparable to that of spontaneous mutagenesis (Ruiter et al., 2003).

Single-stranded DNA oligonucleotides proved to be of a bit higher efficacy (Dong et al., 2006), but it still was not high enough. Moreover, selecting edited plants became a problem that could not be resolved without using vectors. For that reason, the perspectives of this direction remain questionable

Another direction of GE is related to using endonucleases, special enzymes provoking double-stranded ruptures in a DNA molecule. Repairing the ruptures may occur either through recombination with a homologous DNA fragment that has been placed into a vector and transformed into a cell nucleus. The first endonucleases used for this purpose were homing endonucleases recognizing DNA regions of 12–45 nucleotides. The specificity of these regions varied and depended on a type of nuclease, e. g., using the I-CeuI homing endonuclease and the 35S promoter, the bar gene was precisely inserted into a site of a corn genome to make the plant resistant to phosphinothricin (D’Halluin et al., 2008).

Analogous site-specific insertion was carried out in a cotton genome (genus Gossypium) to provide the last with genes hppd and epsps making the plant resistant to glyphosate (D’Halluin et al., 2013). The I-SceI homing endonuclease was used to replace a region in a barley genome to a homologous one delivered in a vector with a functional gene of resistance to hygromycin (Watanabe et al., 2015).

## Protein editors: ZF and TALEN nucleases

In the GE techniques based on protein editing, one uses chimeric nucleases. These are complex proteins containing two structural components, one of which binds specifically with certain nucleotide sequencies of genome DNA, directing at them the second component, a nuclease catalyzing DNA splitting. These proteins are delivered into a plant’s genome using expression vectors

The first such vectors were ZF nucleases that typically contained three “zinc fingers” as a directing structure. The fingers are protein domains binded with one or two ions of zinc and capable of recognizing and specifically binding with a certain nucleotide triplet in DNA sequence. In some case, the number of these domains were increased to 6, so their spe- cificity level raised to 18 DNA nucleotides (Liu et al., 1997).

For the first time, ZF nucleases were applied for genome editing in plants in 2005 when a corresponding vector was inserted in Arabidopsis so indels of different length, mostly deletions (78 %), were found (Lloyd et al., 2005). Since then, a lot of analogous projects have been performed in tabaco, soybean, corn, tomato, apple and fig trees (Shukla et al., 2009; Townsend et al., 2009; Curtin et al., 2011; Peer et al., 2015; Hilioti et al., 2016). However, the technique has turned out to be quite laborious and expensive for it requires a unique protein structure of ZF nuclease to be created for each individual sequence of target DNA. Additionally, the technique is not precise in recognizing nucleotide triplets, which results in a large number of DNA splits in off-target regions. For these reasons, the technique is quite rarely applied these days.

TALEN chimeric nucleases have proved to be more effective. The protein domains serving as their directing structures are the prototypes of the natural TAL effectors of certain bacteria, and each of them recognizes only one nucleotide. In this case, the DNA recognition mechanism is more unambiguous than that of ZF nucleases and allows for relatively easy creation of a structure that specifically recognizes a required DNA sequence. The last is binded with an enzyme splitting the DNA (commonly, Fok I endonuclease) and enables for a theoretically very precise double-stranded rupture within any genome region.

In 2011, the technique was recognized as the most perspective GE approach. By 2017, it had been used to edit 12 plant genomes including those of such domestic plants as rice, wheat, corn, tobacco, barley, potato, sugar cane, soybean, tomato, and of model plants such as Arabidopsis and Brachypodium. In total, in these plants, more than 50 genes have been edited (mostly knocked out) (Malzahn et al., 2017), e. g., to increase bioethanol output in the sugar cane, TALEN nucleases were used to knock out its genes responsible for high lignin content (Jung, Alpeter, 2016). To exclude potato sweetening while storing in cold, vacuolar invertase catalyzing the sucrose splitting into fructose and glucose was knocked out (Clasen et al., 2016). Using the TALEN and CRISPR/ Cas9 approaches it became possible to knock out the alleles of powdery mildew resistant loci in every three subgenomes of allohexaploid common wheat Triticum aestivum L. (genome BAD; 2n = 42) (Wang et al., 2014). To improve the quality of soybean oil, the genes of desaturase enzymes were mutated (Haun et al., 2014).

To facilitate the TALEN technique, a number of software solutions have been developed to search for edited sites, create vector structures and detect off-target sites such as TALENdesigner (http://talen-design.de).

## CRISPR/Cas9: leading GE technique

Unlike the chimeric nucleases, in the CRISPR/Cas9 technology, DNA-recognizing structures are not proteins but short RNAs that, first, are far more precise due to their complementarity and, second, are much easier and chipper to synthesize. The theoretical foundation of the technology was laid while studying the mechanism bacteria use to get protected from pathogenic viruses (bacteriophages) (Savitskaya et al., 2016). There have been published many reviews devoted to CRISPR/Cas9 (Khlestkina, Shumny, 2016; Zlobin et al., 2017; Strygina, Khlestkina, 2020). In plants, the technology was first applied in 2013 (Li et al., 2013; Nekrasov et al., 2013; Shan et al., 2013)

The simplified vector included the genes of the Cas9 protein, a guide RNA (gRNA) analogous to bacterial CRISPR RNA and an additional sequence coding a nuclear localization signal (NLS). The vector was introduced in plant cells using either agrobacterial transformation or bioballistics. As a result, cellular DNA were transcripted by the intercellular RNA polymerase III. From the RNA template encoding Cas9, a protein is translated on ribosomes, which then enters the nucleus via NLS. In the nucleus the gRNA and Cas9 got united to bind with its target site following the principle of complimentary interaction.

An important element that, in many ways, determined the specificity of the binding was a protospacer adjacent motif (PAM), a nucleotide triplet (commonly NGG) placed near the 3′-end of the target site. The catalytic domains of the nuclease provoked single-stranded breaks near the PAM to activate a repair mechanism that could act in two ways: nonhomological end joining (NHEJ) being prone to the errors producing the indels of one or several nucleotides that shift the reading frame of the coded protein and disrupting its functionality to the degree of a knockout. The second way is homology-dependent repair (HDR) that edits the target site or introduces a new sequence that can be undesirable for an experiment, but the last is only possible if such a fragment of donor DNA has already presented in the region being edited

The key element leading to successful genome edition via CRISPR/Cas9 has been selecting a gRNA for a target gene. The site of interaction with gRNA does not usually exceed 30 bp. The presence of PAM at the 3′-end of this region is an important condition for selecting a site to be edited. Another important criterion for gRNA selection is the number and localization of the sites for off-target editing, whose search in a genome is performed individually for each particular gRNA using special software solutions like those available on http:// crispr.mit.edu/.

Lately, the GE technique using ribonucleoprotein (RNP) complexes has been actively developed. In this case, the transforming agent is not a vector (plasmid RNA) but a readyto- use complex including Cas9 and a gRNA. This approach has proved its efficacy when editing the genomes of corn, wheat and potato via bombarding the embryonal cells with gold microparticles (Martin-Ortigosa et al., 2014; Woo et al., 2015; Svitashev et al., 2016; Liang et al., 2017; Andersson et al., 2018).

It is noteworthy that this alternative to using an agrobacterium, which by itself can cause an undesirable genetic effect, allows CRISPR/Cas9 to go beyond the GMO approach and overcome the forbiddance against its application in the agricultural industry. Its other advantage is the reduced likelihood of DNA cutting in off-target sites because the lifetime of a delivered RNP complex is much shorter than its DNA expression. At the same time, employing bioballistics for delivering RNP complexes has a number of drawbacks related to the technique’s excessive traumaticity for plant tissues, complexity of transformation and regeneration, and low editing frequency. For that reason, vector-based agrobacterial transformation still remains a leading approach to CRISPR/Cas9.

## Using CRISPR/Cas9 for producing new cultivars

Genome editing is a technology that can serve both applied – obtaining plants with new useful properties – and fundamental – studying the functions of genes – purposes. The fundamental tasks are solved using the methods of inverted genetics when scientists manipulate genetic sequencies knocking out this or that gene to see what consequences it will cause in the phenotype.

As for applied problems they are quite diverse and in what follows, the main directions of CRISPR/Cas9 application for breeding will be considered.

Resistance to pathogens

The Table displays the studies aimed at creating the plants resistant to different pathogens. For instance, in rice (Oryza sativa L.) applying CRISPR/Cas9 resulted in its resistance to three pathogens: bacterial blight, tungro spherical virus and blast fungus. In the first case, the resistance was achieved after knocking out one of the S genes responsible for sensitivity to bacterial blight (sucrose transportation gene OsSWEET13 being a target for a bacterial TAL effector (Zhou et al., 2015). In the second case, the host’s eIF4G gene was knocked out whose product controlled the initiation of viral RNA translation (Macovei et al., 2018). And finally, in case of fungal pathogen, it was the OsERF922 gene that was knocked out and it led to the reduction in ethylene hormone level in the cells and increased resistance (Wang et al., 2016).

In T. aestivum, fungal pathogen Blumeria graminis f. sp. tritici causes the so-called powdery mildew that significantly reduces the yield of common wheat in many regions. Currently, the S genes responsible for the sensitivity to the fungus have been edited. In one of such studies, the MLO genes were knocked out (Wang et al., 2014), in another – the EDR1 (enhanced disease resistance) genes (Zhang et al., 2017). It has been shown that in both cases, a knockout of all three homoelogical copies of the gene is to be achieved since knocking out only one or two copies has only resulted in partial resistance to the disease.

In Solanum lycopersicum L., application of CRISPR/Cas9 has made it possible to obtain tomato cultivars resistant to bacterial speck, yellow leaf curl virus and powdery mildew. In the first case, to enhance the barrier preventing bacterial infiltration in the cells, the SlJAZ2 gene to control stoma closure was mutated to foster the gain of function (Ortigosa et al., 2018). In the case of viral disease, these were the pathogen’s genes that were targeted, namely, the viral envelope (CP) and replicase (Rep) genes. As a part of T-DNA, their short sequencies were built in the plant’s nuclear genome to enable their constitutive expression as RNA molecules, which in combination with Cas9 could effectively interfere the viral DNA (Tashkandi et al., 2018).

Resistance to abiotic stress

A number of studies aimed at developing the cultivars resistant to abiotic stresses are listed in the Table. For instance, applying the protoplast technique in wheat led to mutating two genes related to drought stress (TaDREB2 and TaERF3) (Kim D. et al., 2017). A similar study was performed in soybean (Glycine max L.) in which two genes related to the plant’s resistance to drought and salinity (Curtin et al., 2018).

In this field, not only applied but also fundamental research has been performed. Hence, it was found out that mitogenicactivated protein kinase (MAPK) reacted to drought by protecting a cell membrane from oxidation and regulating the transcription of other genes. The role of one of MAPK genes was determined using CRISPR/Cas9 for creating the knockout mutants of this gene (Wang et al., 2017). In a similar way, the effect of three genes on rice resistance to abiotic factors was determined. It turned out, the genes coded MAPK (OsMPK2), phytoene desaturase (OsPDS) and betaine aldehyde dehydrogenase (OsBADH2) (Shan et al., 2013).

Yield

The studies applying CRISPR/Cas9 to increase a plant’s yield are listed in the Table. The kernel size and thousand-kernel weight in common wheat were increased by provoking nonsense mutations in the homeological copy of the GW2 gene being a negative regulator of these traits. The degree of the increase was determined by a portion of mutated homoeological genes (Wang W. et al., 2018). Later, the same authors could change the size and weight of a wheat kernel by mutating the sequence of another gene to belong to the same group: GW7 in subgenomes B and D (Wang et al., 2019).

**Table 1.**
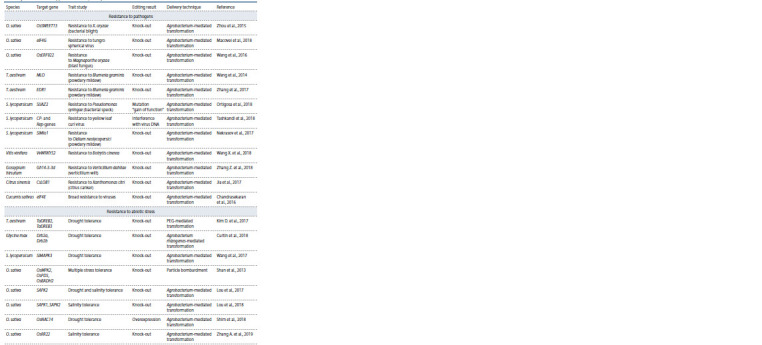
Summary of CRISPR/Cas9 applications in major crops

**Table 1end.**
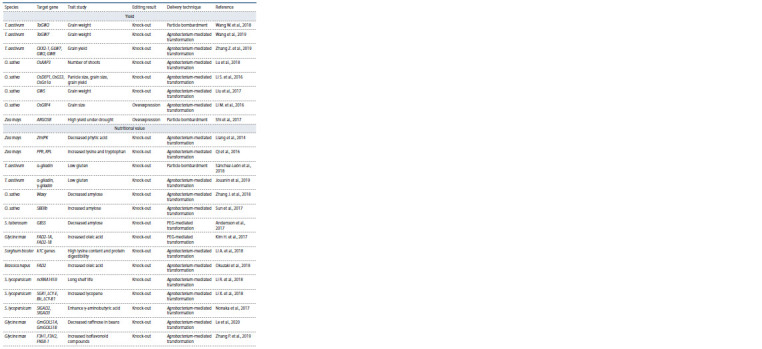
Table 1end

The number of kernels in an ear was increased by editing four target genes: CKX2-1, GLW7, GW2 and GW8 (Zhang A. et al., 2019). In this case, the line homozygotic to the large deletion in the CKX2-1 gene demonstrated the maximum increase of the ear kernel number as well as maximum ear density, which has confirmed the gene is a negative regulator affecting the number of kernels in an ear.

A whole set of genes was knocked out in rice. These were negative regulators of controlling such traits as tiller number (OsAAP3), ear size (OsDEP1), kernel weight (OsGW5) and size (OsGS3, OsGRF4) and the number of kernels in an ear (OsGn1a) (Li M. et al., 2016; Li S. et al., 2016; Liu et al., 2017; Lu et al., 2018). Additionally, the rice model has been applied to integrate whole-genome sequencing, genealogy analysis and CRISPR/Cas9 for full-scale identification of the target genes that affect quantitative traits including yield (Huang et al., 2018).

At the first stage, the genealogy analysis detected multiple quantitative trait loci (QTL) associated with yield to carry out their association mapping. Comparison of the obtained map against the rice’s whole-genome sequence enabled for selecting candidate genes to be knocked out using CRISPR/ Cas9 for estimating their phenotypical effect. As a result, a whole set of the genes crucial for yield was found.

A study to preserve the yield in presence of stress factors by mutating the ARGOS8 gene was carried out in corn (Zea mays L.) by J. Shi et al. (2017). The authors applied CRISPR/ Cas9 to replace this negative regulator of ethylene response by a promotor of another gene to increase ARGOS8 expression. Field studied have demonstrated that the CRISPRedited plants had higher yield in drought condition than their parents.

Nutritional value

High amounts of phytic acid present in the grains of cereal, legume and oil crops. This acid is antinutrient and cannot be digested by animals with single-chamber stomach and can cause environmental pollution. To reduce the acid’s content in corn, CRISPR/Cas9 was applied to knock out the gene of the enzyme catalyzing the stages of phytic-acid biosynthesis, so its production was blocked in the mutant line (Liang et al., 2014). The same corn was used to obtain cultivars with higher level of essential amino acids – lysin and tryptophane – by knocking out the genes having a negative effect on their biosynthesis (Qi et al., 2016).

Changing gluten content and composition in wheat has been another topical issue due to the high spread of gluten intolerance in people. The results of two studies using CRISPR/Cas9 and aimed at reducing in wheat the content of α- and γ-gliadins causing pathological reactions have recently been published. One group obtained the mutant lines with significantly reduced α-gliadin content (Sánchez-León et al., 2018). The other group created lines with low α- and γ-gliadins (Jouanin et al., 2019). The obtained wheat lines may become a start for new elite wheat cultivars to produce low-gluten products.

In rice, application of CRISPR/Cas9 has led to the plant’s improved nutritive and culinary qualities. It was achieved by mutating the Waxy gene to change the amylose/amylopectin ratio in starch in the favor of amylopectin (Zhang J. et al., 2018). This component determines the waxlike (sticky) qualities of starch in rice grains, which is very important for making sushi. In another study, the opposite result was obtained, so the gene responsible for suppressing amylose synthesis was knocked out (Sun et al., 2017).

In potato (Solanum tuberosum L.), the gene encoding granule-bound starch synthase (GBSS) was knocked out, so the obtained lines demonstrated a reduced level of amylose (Andersson et al., 2017).

To improve the quality of soybean oil, CRISPR/Cpf1 was used to knock out genes FAD2-1B and FAD2-1A and produce high-yield soy plants with high content of oleic acid (Kim H. et al., 2017).

In sorgo (Sorghum bicolor L.), GE techniques were applied to knock out the genes responsible for improper digestibility and essential amino acids suppression (Li A. et al., 2018).

Using CRISPR/Cas9 the cultivars of rape (Brassica napus L.) were obtained with high content of oleic acid (Okuzaki et al., 2018) as well tomato cultivars with increased storability (Li R. et al., 2018) and increased content of lycopene, a vitamin A precursor of powerful antioxidation effect (Li X. et al., 2018). These and many other studies are listed in the Table.

De novo domestication

The essence of the de novo domestication approach is speeding up a domestication process for a wild relative of an agricultural plant. The wild relatives are widely used in selection as donors of the genes responsible for a plant’s resistance to biotic and abiotic stresses. However, a simple crossing with a wild species only produces ‘half-cultivars’ that often lose the features of a cultural plant as well as the many qualities useful for humans

Studies into the genes of wild and domestic plants have found the so-called ‘domestication genes’, in other words, mutations that transform a wild plant into one applicable for farming.

The idea behind de novo domestication is dedicated introduction of necessary genes into the domestication genes of a cultural plant’s wild relative. Such boosted domestication made the headlines in 2018, when CRISPR/Cas9 was applied to convert a wild tomato into an almost cultural plant in a singe generation. To do so, a list of genes to be modified to obtain the plant’s de novo version had been composed (Zsögön et al., 2017).

Comparing the genetic sequences in both wild and cultural tomato enabled one to determine the structural modifications to be implemented in the wild plant. At the final stage of the experiment, multiplex editing of four genes (SP, SP5G, SlCLV3 and SlWUS was performed. These genes controlled the plant’s architecture (transition to the determinate type), heading time and fruit size (Li T. et al., 2018).

Another example of such research is changing the morphology of a barley ear. The naked kernel, unlike the rough one, has always been a sign of the crop’s domestication. Naked barley is a traditional food and currently considered as a dietary component of functional nutrition. In nature, this transition from chuffy to naked kernel was determined by the NUD gene losing its function due to deletion of 17 kb in a corresponding locus. Using CRISPR/Cas9, a naked-barley cultivar has been produced experimentally by knocking out NUD in a wild rough variety (Gerasimova et al., 2020).

Thus, de novo domestication opens huge perspectives for selective breeding, enabling one to obtain the results of hundreds and thousands of years of evolution in one generation

## Conclusion

Intensely developing GE technologies will soon see lifting many of the limitations for their wide practical application. The development goes in the direction of higher modification specificity and off-target effects elimination by using new-type nucleases such as the Cas9 orthologs interacting with different PAMs (Fonfara et al., 2014) or completely new nucleases such as Cas12a (Zetsche et al., 2015).

Moreover, there are approaches that go beyond gene knockouts and include other modifications as changing a nucleotide or a whole sequence. This method has proved effective when editing a single DNA base to perform cytosine/thymine or adenine/guanine replacement. Such changes have become possible thanks to using specific enzymes being a combination of cytosine deaminase, adenosine desaminase and nickase (Zong et al., 2017; Li C. et al., 2018).

Another technique that is developing fast is homological recombination when an expressing vector is delivered in a cell together with a donor DNA flankered by the sequencies homologous to the site where endogenous DNA is replaced by a donor’s one (Jasin, Haber, 2016).

In addition, transformation techniques are developing since the classical methods such as agrobacterial transformation and particle bombardment in many ways produce low output of transformants. Hence, a possibility to use modified viral genomes has been demonstrated for transition of expression cassettes, geminiviruses in particular, and proved effective for a number of cultures (Baltes et al., 2014; Čermák et al., 2015; Butler et al., 2016).

Along with technological advancements, the development of bioinformatic approaches, in particular, enlargement of genetic databases and enhancing of genetic network analysis will become the basis for multiplex GE to modify several traits at once.

## Conflict of interest

The authors declare no conflict of interest.

## References

Ahloowalia B.S., Maluszynski M., Nichterlein K. Global impact of
mutation-derived varieties. Euphytica. 2004;135(2):187-204. DOI
10.1023/B:EUPH.0000014914.85465.4f.

Andersson M., Turesson H., Nicolia A., Fält A.S., Samuelsson M.,
Hofvander P.E. Efficient targeted multiallelic mutagenesis in tetraploid
potato (Solanum tuberosum) by transient CRISPR-Cas9 expression
in protoplasts. Plant Cell Rep. 2017;36(1):117-128. DOI
10.1007/s00299-016-2062-3

Andersson M., Turesson H., Olsson N., Fält A.-S., Ohlsson P., Gonzalez
M.N., Samuelsson M., Hofvander P. Genome editing in potato
via CRISPR-Cas9 ribonucleoprotein delivery. Physiol. Plant. 2018;
164(4):378-384. DOI 10.1111/ppl.12731.

Bak R.O., Gomez-Ospina N., Porteus M.H. Gene editing on center
stage. Trends Genet. 2018;34(8):600-611. DOI 10.1016/j.tig.2018.
05.004.

Baltes N.J., Gil-Humanes J., Cermak T., Atkins P.A., Voytas D.F. DNA
replicons for plant genome engineering. Plant Cell. 2014;26(1):
151-163. DOI 10.1105/tpc.113.119792.

Banerjee S.K., Borden A., Christensen R.B., LeClerc J.E., Lawrence
C.W. SOS-dependent replication past a single trans-syn T-T
cyclobutane dimer gives a different mutation spectrum and increased
error rate compared with replication past this lesion in uniduced cell.
J. Bacteriol. 1990;172(4):2105-2112. DOI 10.1128/jb.172.4.2105-
2112.1990.

Beetham P.R., Kipp P.B., Sawycky X.L., Arntzen C.J., May G.D. A tool
for functional plant genomics: chimeric RNA/DNA oligonucleotides
cause in vivo gene-specific mutations. Proc. Natl. Acad. Sci. USA.
1999;96(15):8774-8778. DOI 10.1073/pnas.96.15.8774.

Burkhardt P.K., Beyer P., Wünn J., Klöti A., Armstrong G.A.,
Schledz M., von Lintig J., Potrykus I. Transgenic rice (Oryza sativa)
endosperm expressing daffodil (Narcissus pseudonarcissus) phytoene
synthase accumulates phytoene, a key intermediate of provitamin
A biosynthesis. Plant J. 1997;11(5):1071-1078. DOI 10.1046/
j.1365-313x.1997.11051071.x.

Butler N.M., Baltes N.J., Voytas D.F., Douches D.S. Geminivirusmediated
genome editing in potato (Solanum tuberosum L.) using
sequence-specific nucleases. Front. Plant Sci. 2016;7:1045. DOI
10.3389/fpls.2016.01045

Capecchi M.R. Altering the genome by homologous recombination.
Science. 1989;244(4910):1288-1292. DOI 10.1126/science.2660260

Čermák T., Baltes N.J., Čegan R., Zhang Y., Voytas D.F. High-frequency,
precise modification of the tomato genome. Genome Biol. 2015;
16:232. DOI 10.1186/s13059-015-0796-9.

Chandrasekaran J., Brumin M., Wolf D., Leibman D., Klap C., Pearlsman
M., Sherman A., Arazi T., Gal-On A. Development of broad
virus
resistance in non-transgenic cucumber using CRISPR/Cas9
technology. Mol. Plant Pathol. 2016;17(7):1140-1153. DOI 10.1111/
mpp.12375.

Cherny I.V. Novosibirskaya-67 common wheat radiation variety: creation
and introduction into agricultural production in West Siberia.
Novosibirsk: Institute of Cytology and Genetics, 1982. (in Russian)

Clasen B.M., Stoddard T.J., Luo S., Demorest Z.L., Li J., Cedrone F.,
Tibebu R., Davison S., Ray E.E., Daulhac A., Coffman A., Yabandith
A., Retterath A., Haun W., Baltes N.J., Mathis L., Voytas D.F.,
Zhang F. Improving cold storage and processing traits in potato
through targeted gene knockout. Plant Biotechnol. J. 2016;14(1):
169-176. DOI 10.1111/pbi.12370

Curtin S.J., Xiong Y., Michno J.M., Campbell B.W., Stec A.O., Čermák
T., Starker C., Voytas D.F., Eamens A.L., Stupar R.M. CRISPR/
Cas9 and TALENs generate heritable mutations for genes involved
in small RNA processing of Glycine max and Medicago truncatula.
Plant Biotechnol. J. 2018;16(6):1125-1137. DOI 10.1111/pbi.
12857.

Curtin S.J., Zhang F., Sander J.D., Haun W.J., Starker C., Baltes N.J.,
Reyon D., Dahlborg E.J., Goodwin M.J., Coffman A.P., Dobbs D.,
Joung J.K., Voytas D.F., Stupar R.M. Targeted mutagenesis of duplicated
genes in soybean with zinc-finger nucleases. Plant Physiol.
2011;156(2):466-473. DOI 10.1104/pp.111.172981.

D’Halluin K., Vanderstraeten C., Hulle J., Rosolowska J., Den Brande I.,
Pennewaert A., D’Hont K., Bossut M., Jantz D., Ruiter R., Broadhvest
J. Targeted molecular trait stacking in cotton through targeted double-strand break induction. Plant Biotechnol. J. 2013;11(8):933-
941. DOI 10.1111/pbi.12085

D’Halluin K., Vanderstraeten C., Stals E., Cornelissen M., Ruiter R.
Homologous recombination: a basis for targeted genome optimization
in crop species such as maize. Plant Biotechnol. J. 2008;6(1):
93-102. DOI 10.1111/j.1467-7652.2007.00305.x.

Dong C., Beetham P., Vincent K., Sharp P. Oligonucleotide-directed
gene repair in wheat using a transient plasmid gene repair assay
system. Plant Cell Rep. 2006;25(5):457-465. DOI 10.1007/s00299-
005-0098-x.

Dudin M.N. Transgenic organisms (GMOs) in agriculture: an objective
neeed to ensure global food security or a way to increase the profits
of TNCs in agro-industrial complex? Prodovolstvennaya Politika i
Bezopasnost = Food Policy and Security. 2020;7(2):107-120. DOI
10.18334/ppib.7.2.100666. (in Russian)

Fonfara I., Le Rhun A., Chylinski K., Makarova K.S., Lécrivain A.L.,
Bzdrenga J., Koonin E.V., Charpentier E. Phylogeny of Cas9 determines
functional exchangeability of dual-RNA and Cas9 among
orthologous type II CRISPR-Cas systems. Nucleic Acids Res. 2014;
42(4):2577-2590. DOI 10.1093/nar/gkt1074.

Gaud W.S. The Green Revolution: Accomplishments and Apprehensions.
1968. No. REP-11061. CIMMYT. www.agbioworld.org.

Genetically Engineered Crops: Experiences and Prospects. Washington
(DC): National Academies Press, 2016. DOI 10.17226/23395.

Gerasimova S., Hertig C., Korotkova A.M., Kolosovskaya E.V., Otto I.,
Hiekel S., Kochetov A.V., Khlestkina E.K., Kumlehn J. Conversion
of hulled into naked barley by Cas endonuclease-mediated knockout
of the NUD gene. BMC Plant Biol. 2020;20(Suppl. 1):255. DOI
10.1186/s12870-020-02454-9.

Guirouilh-Barbat J., Huck S., Bertrand P., Pirzio L., Desmaze C., Sabatier
L., Lopez B.S. Impact of the KU80 pathway on NHEJ-induced
genome rearrangements in mammalian cells. Mol. Cell. 2004;14(5):
611-623. DOI 10.1016/j.molcel.2004.05.008

Hall B., Limaye A., Kulkarni A. Overview: generation of gene knockout
mice. Curr. Protoc. Cell Biol. 2009;19(1):19.12.1-19.12.17. DOI
10.1002/0471143030.cb1912s44.

Haun W., Coffman A., Clasen B.M., Demorest Z.L., Lowy A., Ray E.,
Retterath A., Stoddard T., Juillerat A., Cedrone F., Mathis L., Voytas
D.F., Zhang F. Improved soybean oil quality by targeted mutagenesis
of the fatty acid desaturase 2 gene family. Plant Biotechnol.
J. 2014;12(7):934-940. DOI 10.1111/pbi.12201

Hilioti Z., Ganopoulos I., Ajith S., Bossis I., Tsaftaris A. A novel arrangement
of zinc finger nuclease system for in vivo targeted genome
engineering: the tomato LEC1-LIKE4 gene case. Plant Cell
Rep. 2016;35(11):2241-2255. DOI 10.1007/s00299-016-2031-x.

Huang J., Li J., Zhou J., Wang L., Yang S., Hurst L.D., Li W.-H.,
Tian D. Identifying a large number of high-yield genes in rice by
pedigree analysis, whole-genome sequencing, and CRISPR-Cas9
gene knockout. Proc. Natl. Acad. Sci. USA. 2018;115(32):E7559-
E7567. DOI 10.1073/pnas.1806110115.

Jasin M., Haber J.E. The democratization of gene editing: Insights from
site-specific cleavage and double-strand break repair. DNA Repair.
2016;44:6-16. DOI 10.1016/j.dnarep.2016.05.001.

Jia H., Zhang Y., Orbović V., Xu J., White F.F., Jones J.B., Wang N.
Genome editing of the disease susceptibility gene CsLOB1 in citrus
confers resistance to citrus canker. Plant Biotechnol. J. 2017;15(7):
817-823. DOI 10.1111/pbi.12677.

Jonczyk P., Fijalkowska I., Ciesla Z. Overproduction of the subunit of
DNA polymerase III counteracts the SOS-mutagenic response of
Eischerichia coli. Proc. Natl. Acad. Sci. USA. 1988;85(23):2124-
2127. DOI 10.1073/pnas.85.23.9124.

Jouanin A., Schaart J.G., Boyd L.A., Cockram J., Leigh F.J., Bates R.
Outlook for coeliac disease patients: towards bread wheat with
hypoimmunogenic
gluten by gene editing of α- and γ-gliadin gene
families. BMC Plant Biol. 2019;19:333. DOI 10.1186/s12870-019-
1889-5.

Jung J.H., Altpeter F. TALEN mediated targeted mutagenesis of the
caffeic acid O-methyltransferase in highly polyploid sugarcane
improves cell wall composition for production of bioethanol. Plant
Mol. Biol. 2016;92(1-2):131-142. DOI 10.1007/s11103-016-0499-y.

Karthik K., Nandiganti M., Thangaraj A., Singh S., Mishra P., Rathinam
M., Sharma M., Singh N.K., Dash P.K., Sreevathsa R. Transgenic
cotton (Gossypium hirsutum L.) to combat weed vagaries: utility
of an apical meristem-targeted in planta transformation strategy
to introgress a modified CP4-EPSPS gene for glyphosate tolerance.
Front. Plant Sci. 2020;11:768. DOI 10.3389/fpls.2020.00768.

Khlestkina E.K., Shumny V.K. Prospects for application of breakthrough
technologies in breeding: The CRISPR/Cas9 system for
plant genome editing. Russ. J. Genet. 2016;52(7):676-687. DOI
10.1134/S102279541607005X.

Khush G.S. Genetically modified crops: the fastest adopted crop technology
in the history of modern agriculture. Agric. Food Secur.
2012;1:14. DOI 10.1186/2048-7010-1-14.

Kilian B., Mammen K., Millet E., Sharma R., Graner A., Salamini F.,
Hammer K., Özkan H. Aegilops. In: Kole C. (Ed.). Wild Crop Relatives:
Genomic and Breeding Resources. Berlin: Springer, 2011;
1-76. DOI 10.1007/978-3-642-14228-4_1.

Kim D., Alptekin B., Budak H. CRISPR/Cas9 genome editing in wheat.
Funct. Integr. Genomics. 2017;18(1):31-41. DOI 10.1007/s10142-
017-0572-x.

Kim H., Kim S.T., Ryu J., Kang B.C., Kim J.S., Kim S.G. CRISPR/
Cpf1-mediated DNA-free plant genome editing. Nat. Commun.
2017;8:14406. DOI 10.1038/ncomms14406.

Kochevenko A., Willmitzer L. Chimeric RNA/DNA oligonucleotidebased
site-specific modification of the tobacco acetolactate syntase
gene. Plant Physiol. 2003;132(1):174-184. DOI 10.1104/pp.102.
016857.

König A., Cockburn A., Crevel R.W., Debruyne E., Grafstroem R.,
Hammerling U., Kimber I., Knudsen I., Kuiper H.A., Peijnenburg
A.A., Penninks A.H., Poulsen M., Schauzu M., Wal J.M. Assessment
of the safety of foods derived from genetically modified
(GM) crops. Food Chem. Toxicol. 2004;42(7):1047-1088. DOI
10.1016/j.fct.2004.02.019.

Le H., Nguyen N.H., Ta D.T., Le T.N.T., Bui T.P., Le N.T., Bui T.P.,
Le N.T., Nguyen C.X., Rolletschek H., Stacey G., Stacey M.G.,
Pham N.B., Do P.T., Chu H.H. CRISPR/Cas9-mediated knockout
of galactinol synthase-encoding genes reduces raffinose family oligosaccharide.
Front. Plant Sci. 2020;11:612942. DOI 10.3389/fpls.
2020.612942.

Li A., Jia S., Yobi A., Ge Z., Sato S., Zhang C., Angelovici R., Clemente
T.E., Holding D.R. Editing of an alpha-kafirin gene family
increases digestibility and protein quality in sorghum. Plant Physiol.
2018;177(4):1425-1438. DOI 10.1104/pp.18.00200.

Li C., Zong Y., Wang Y., Jin S., Zhang D., Song Q., Zhang R., Gao C.
Expanded base editing in rice and wheat using a Cas9-adenosine
deaminase fusion. Genome Biol. 2018;19:59. DOI 10.1186/s13059-
018-1443-z.

Li J., Aach J., Norville J.E., Mccormack M., Bush J., Church G.M.,
Sheen J. Multiplex and homologous recombination-mediated plant
genome editing via guide RNA/Cas9. Nat. Biotechnol. 2013;31(8):
688-691. DOI 10.1038/nbt.2654.

Li M., Li X., Zhou Z., Wu P., Fang M., Pan X., Lin Q., Luo W., Wu G.,
Li H. Reassessment of the four yield-related genes Gn1a, DEP1,
GS3, and IPA1 in rice using a CRISPR/Cas9 system. Front. Plant
Sci. 2016;7:377. DOI 10.3389/fpls.2016.00377.

Li R., Fu D., Zhu B., Luo Y., Zhu H. CRISPR/Cas9-mediated mutagenesis
of lncRNA1459 alters tomato fruit ripening. Plant J. 2018;
94(3):513-524. DOI 10.1111/tpj.13872.

Li S., Gao F., Xie K., Zeng X., Cao Y., Zeng J., He Z., Ren Y., Li W.,
Deng Q., Wang S., Zheng A., Zhu J., Liu H., Wang L., Li P. The
OsmiR396c-OsGRF4-OsGIF1 regulatory module determines grain
size and yield in rice. Plant Biotechnol. J. 2016;14(11):2134-2146.
DOI 10.1111/pbi.12569.

Li T., Yang X., Yu Y., Si X., Zhai X., Zhang H., Dong W., Gao C., Xu C.
Domestication of wild tomato is accelerated by genome editing. Nat.
Biotechnol. 2018;36:1160-1163. DOI 10.1038/nbt.4273.

Li X., Wang Y., Chen S., Tian H., Fu D., Zhu B., Luo Y., Zhu H. Lycopene
is enriched in tomato fruit by CRISPR/Cas9-mediated multiplex
genome editing. Front. Plant Sci. 2018;9:559. DOI 10.3389/
fpls.2018.00559.

Liang Z., Chen K., Li T., Zhang Y., Wang Y., Zhao Q., Liu J., Zhang H.,
Liu C., Ran Y., Gao C. Efficient DNA-free genome editing of bread
wheat using CRISPR/Cas9 ribonucleoprotein complexes. Nat. Commun.
2017;8:14261. DOI 10.1038/ncomms14261.

Liang Z., Zhang K., Chen K., Gao C. Targeted mutagenesis in Zea mays
using TALENs and the CRISPR/Cas system. J. Genet. Genomics.
2014;41(2):63-68. DOI 10.1016/j.jgg.2013.12.001.

Liu J., Chen J., Zheng X., Wu F., Lin Q., Heng Y., Tian P., Cheng Z.,
Yu X., Zhou K., Zhang X., Guo X., Wang J., Wang H., Wan J. GW5
acts in the brassinosteroid signalling pathway to regulate grain
width and weight in rice. Nat. Plants. 2017;3:17043. DOI 10.1038/
nplants.2017.43

Liu Q., Segal D.J., Ghiara J.B., Barbas C.F. Design of polydactyl zincfinger
proteins for unique addressing within complex genomes.
Proc. Natl. Acad. Sci. USA. 1997;94(11):5525-5530. DOI 10.1073/
pnas.94.11.5525.

Lloyd A., Plaisier C.L., Carroll D., Drews G.N. Targeted mutagenesis
using zinc-finger nucleases in Arabidopsis. Proc. Natl. Acad. Sci.
USA. 2005;102(6):2232-2237. DOI 10.1073/pnas.0409339102.

Lou D., Wang H., Liang G., Yu D. OsSAPK2 confers abscisic acid
sensitivity and tolerance to drought stress in rice. Front. Plant Sci.
2017;8:993. DOI 10.3389/fpls.2017.00993.

Lou D., Wang H., Yu D. The sucrose non-fermenting-1-related protein
kinases SAPK1 and SAPK2 function collaboratively as positive regulators
of salt stress tolerance in rice. BMC Plant Biol. 2018;18(1):
203. DOI 10.1186/s12870-018-1408-0.

Lu K., Wu B., Wang J., Zhu W., Nie H., Qian J., Huang W., Fang Z.
Blocking amino acid transporter OsAAP3 improves grain yield by
promoting outgrowth buds and increasing tiller number in rice. Plant
Biotechnol. J. 2018;16(10):1710-1722. DOI 10.1111/pbi.12907.

Lundmark K. Genetically modified maize. BioScience. 2007;57(11):
996. DOI 10.1641/B571115

Macovei A., Sevilla N.R., Cantos C., Jonson G.B., Slamet-Loedin I.,
Čermák T., Voytas D.F., Choi I.-R., Chadha-Mohanty P. Novel alleles
of rice eIF4G generated by CRISPR/Cas9-targeted mutagenesis
confer resistance to Rice tungro spherical virus. Plant Biotechnol.
J. 2018;16:1918-1927. DOI 10.1111/pbi.12927.

Malzahn A., Lowder L., Qi Y. Plant genome editing with TALEN
and CRISPR. Cell Biosci. 2017;7:21. DOI 10.1186/s13578-017-
0148-4.

Martin-Ortigosa S., Peterson D.J., Valenstein J.S., Lin V.S.-Y., Trewyn
B.G., Lyznik L.A., Wang K. Mesoporous silica nanoparticlemediated
intracellular Cre protein delivery for maize genome editing
via loxP site excision. Plant Physiol. 2014;164(2):537-547. DOI
10.1104/pp.113.233650.

Marvier M., McCreedy C., Regetz J., Kareiva P. A meta-analysis of
effects of Bt cotton and maize on nontarget invertebrates. Science.
2007;316(5830):1475-1477. DOI 10.1126/science.1139208.

Muller H.J. Artificial transmutation of the gene. Science. 1927;
66(1699):84-87. DOI 10.1126/science.66.1699.84.

Nadson G., Philippov G. Influence des rayons X sur la sexualité et la
formation des mutantes chez les Champignons inférieurs (Mucorinees).
Comptes Rendues des Séances de la Société de Biologie.
1925;93(2):473-475.

Nekrasov V., Staskawicz B., Weigel D., Jones J.D.G., Kamoun S. Targeted
mutagenesis in the model plant Nicotiana benthamiana using
Cas9 RNA-guided endonuclease. Nat. Biotechnol. 2013;31(8):691-
693. DOI 10.1038/nbt.2655

Nekrasov V., Wang C., Win J., Lanz C., Weigel D., Kamoun S. Rapid
generation of a transgene-free powdery mildew resistant tomato by
genome deletion. Sci. Rep. 2017;7:482. DOI 10.1038/s41598-017-
00578-x.

Nonaka S., Arai C., Takayama M., Matsukura C., Ezura H. Efficient
increase of γ-aminobutyric acid (GABA) content in tomato fruits by
targeted mutagenesis. Sci. Rep. 2017;7:7057. DOI 10.1038/s41598-
017-06400-y.

Okuzaki A., Ogawa T., Koizuka C., Kaneko K., Inaba M., Imamura J.,
Koizuka N. CRISPR/Cas9-mediated genome editing of the fatty
acid desaturase 2 gene in Brassica napus. Plant Physiol. Biochem.
2018;131:63-69. DOI 10.1016/j.plaphy.2018.04.025.

Okuzaki A., Toriyama K. Chimeric RNA/DNA oligonucleotide-directed
gene targeting in rice. Plant Cell Rep. 2004;22(7):509-512.
DOI 10.1007/s00299-003-0698-2.

Ortigosa A., Gimenez-Ibanez S., Leonhardt N., Solano R. Design of a
bacterial speck resistant tomato by CRISPR/Cas9-mediated editing
of SlJAZ2. Plant Biotechnol. J. 2018;17(3):665-673. DOI 10.1111/
pbi.13006.

Peer R., Rivlin G., Golobovitch S., Lapidot M., Gal-On A., Vainstein A.,
Tzfira T., Flaishman M.A. Targeted mutagenesis using zinc-finger
nucleases in perennial fruit trees. Planta. 2015;241(4):941-951. DOI
10.1007/s00425-014-2224-x.

Qi W., Zhu T., Tian Z., Li C., Zhang W., Song R. High-efficiency
CRISPR/
Cas9 multiplex gene editing using the glycine tRNA-processing
system-based strategy in maize. BMC Biotechnol. 2016;
16:58. DOI 10.1186/s12896-016-0289-2.

Rapoport I.A. Carbonyl compounds and the chemical mechanism of
mutations. Doklady AN SSSR = Proceedings of the Academy of Sciences
of the USSR. 1946;54:65-68. (in Russian)

Ruiter R., Van Den Brande I., Stals E., Delaure S., Cornelissen M.,
D’Halluin K. Spontaneous mutation frequency in plants obscures
the effect of chimeraplasty. Plant Mol. Biol. 2003;53(5):715-729.
DOI 10.1023/B:PLAN.0000019111.96107.01.

Russian Sun Flower. Krasnodar: Sovetskaya Kuban Publ., 2007. (in
Russian)

Sakuraba Y., Sezutsu H., Takahasi K.R., Tsuchihashi K., Ichikawa R.,
Fujimoto N., Kaneko S., Nakai Y., Uchiyama M., Goda N., Motoi
R., Ikeda A., Karashima Y., Inoue M., Kaneda H., Masuya H.,
Minowa O., Noguchi H., Toyoda A., Sakaki Y., Wakana S., Noda T.,
Shiroishi T., Gondo Y. Molecular characterization of ENU mouse
mutagenesis and archives. Biochem. Biophys. Res. Commun. 2005;
336(2):609-616. DOI 10.1016/j.bbrc.2005.08.134.

Sánchez-León S., Gil-Humanes J., Ozuna C.V., Giménez M.J., Sousa
C., Voytas D.F. Low-gluten, nontransgenic wheat engineered
with CRISPR/Cas9. Plant Biotechnol. J. 2018;16(4):902-910. DOI
10.1111/pbi.12837.

Sapehin A.A. Röntgen-Mutationen beim Weizen (Triticum vulgare).
Der Züchter. 1930;2:257-259.

Savitskaya E.E., Musharova O.S., Severinov K.V. Diversity of
CRISPR-Cas-mediated mechanisms of adaptive immunity in prokaryotes
and their application in biotechnology. Biochemistry (Moscow).
2016;81(7):653-661. DOI 10.1134/S0006297916070026.

Schütte G., Eckerstorfer M., Rastelli V., Reichenbecher W., Restrepo-
Vassalli S., Ruohonen-Lehto M., Saucy A.-G.W., Mertens M. Herbicide
resistance and biodiversity: agronomic and environmental
aspects of genetically modified herbicide-resistant plants. Environ.
Sci. Eur. 2017;29(1):5. DOI 10.1186/s12302-016-0100-y.

Shan Q., Wang Y., Li J., Zhang Y., Chen K., Liang Z., Zhang K., Liu J.,
Xi J.J., Qiu J.-L., Gao C. Targeted genome modification of crop
plants using a CRISPR-Cas system. Nat. Biotechnol. 2013;31(8):
686-688. DOI 10.1038/nbt.2650.

Shi J., Gao H., Wang H., Lafitte H.R., Archibald R.L., Yang M., Hakimi
S.M., Mo H., Habben J.E. ARGOS8 variants generated by
CRISPR-Cas9 improve maize grain yield under field drought stress
conditions. Plant Biotechnol. J. 2017;15(2):207-216. DOI 10.1111/
pbi.12603.

Shim J.S., Oh N., Chung P.J., Kim Y.S., Choi Y.D., Kim J.K. Overexpression
of OsNAC14 improves drought tolerance in rice. Front.
Plant Sci. 2018;9:310. DOI 10.3389/fpls.2018.00310.

Shukla V.K., Doyon Y., Miller J.C., DeKelver R.C., Moehle E.A., Worden
S.E., … Gregory P.D., Urnov F.D. Precise genome modification
in the crop species Zea mays using zinc-finger nucleases. Nature.
2009;459(7245):437-441. DOI 10.1038/nature07992.

Smithies O., Gregg R.G., Boggs S.S., Koralewski M.A., Kucherlapati
R.S. Insertion of DNA sequences into the human chromosomal
β-globin locus by homologous recombination. Nature. 1985;
317(6034):230-234. DOI 10.1038/317230a0.

Strygina K.V., Khlestkina E.K. Wheat, barley and maize genes editing
using the CRISPR/Cas system. Biotekhnologiya i Selektsiya Rasteniy
= Biotechnology and Plant Breeding. 2020;3(1):46-55. DOI
10.30901/2658-6266-2020-1-o2. (in Russian)

Stubbe H. Mutanten der Kulturtomate Lycopersicon esculentum Miller
I. Die Kulturpf lanze. 1957;5:190-220. DOI 10.1007/BF0209
5495.

Sun Y., Jiao G., Liu Z., Zhang X., Li J., Guo X., Du W., Du J., Francis
F., Zhao Y., Xia L. Generation of high-amylose rice through
CRISPR/ Cas9-mediated targeted mutagenesis of starch branching enzymes.
Front. Plant Sci. 2017;8:298. DOI 10.3389/fpls.2017.00298

Sur S., Pagliarini R., Bunz F., Rago C., Diaz L.A., Kinzler K.W., Vogelstein
B., Papadopoulosa N. A panel of isogenic human cancer cells
suggests a therapeutic approach for cancers with inactivated p53.
Proc. Natl. Acad. Sci. USA. 2009;106(10):3964-3969. DOI 10.1073/
pnas.0813333106.

Svitashev S., Schwartz C., Lenderts B., Young J.K., Cigan A.M. Genome
editing in maize directed by CRISPR-Cas9 ribonucleoprotein
complexes. Nat. Commun. 2016;7:13274. DOI 10.1038/ncomms
13274.

Tan S., Evans R.R., Dahmer M.L., Singh B.K., Shaner D.L. Imidazolinone-
tolerant crops: history, current status and future. Pest Manag.
Sci. 2005;61(3):246-257. DOI 10.1002/ps.993.

Tashkandi M., Ali Z., Aljedaani F., Shami A., Mahfouz M.M. Engineering
resistance against Tomato yellow leaf curl virus via the
CRISPR/ Cas9 system in tomato. Plant Signal. Behav. 2018;13(10):
e1525996. DOI 10.1080/15592324.2018.1525996.

Timofeeff-Ressovsky N.W. The effect of X-rays in producing somatic
genovariations of a definite locus in different directions in Drosophila
melanogaster. Am. Nat. 1929;63(685):118-124.

Townsend J.A., Wright D.A., Winfrey R.J., Fu F., Maeder M.L.,
Joung J.K., Voytas D.F. High frequency modification of plant genes
using engineered zinc finger nucleases. Nature. 2009;459:442-445.
DOI 10.1038/nature07845.

Wang F., Wang C., Liu P., Lei C., Hao W., Gao Y., Liu Y.-G., Zhao K.
Enhanced rice blast resistance by CRISPR/Cas9-targeted mutagenesis
of the ERF transcription factor gene OsERF922. PLoS One.
2016;11(4):e0154027. DOI 10.1371/journal.pone.0154027.

Wang L., Chen L., Li R., Zhao R., Yang M., Sheng J., Shen L. Reduced
drought tolerance by CRISPR/Cas9-mediated SlMAPK3 mutagenesis
in tomato plants. J. Agric. Food Chem. 2017;65(39):8674-
8682. DOI 10.1021/acs.jafc.7b02745.

Wang W., Pan Q., Tian B., He F., Chen Y., Bai G., Akhunova A.,
Trick H.N., Akhunov E. Gene editing of the wheat homologs of
TONNEAU1-recruiting motif encoding gene affects grain shape
and weight in wheat. Plant J. 2019;100(2):251-264. DOI 10.1111/
tpj.14440.

Wang W., Simmonds J., Pan Q., Davidson D., He F., Battal A., Akhunova
A., Trick H.N., Uauy C., Akhunov E. Gene editing and mutagenesis
reveal inter-cultivar differences and additivity in the contribution
of TaGW2 homoeologues to grain size and weight in wheat.
Theor. Appl. Genet. 2018;131(11):2463-2475. DOI 10.1007/s00122-
018-3166-7.

Wang X., Tu M., Wang D., Liu J., Li Y., Li Z., Wang Y., Wang X.
CRISPR/
Cas9-mediated efficient targeted mutagenesis in grape in
the first generation. Plant Biotechnol. J. 2018;16(4):844-855. DOI
10.1111/pbi.12832.

Wang Y., Cheng X., Shan Q., Zhang Y., Liu J., Gao C., Qiu J.L. Simultaneous
editing of three homoeoalleles in hexaploid bread wheat
confers heritable resistance to powdery mildew. Nat. Biotechnol.
2014;32(9):947-951. DOI 10.1038/nbt.2969.

Watanabe K., Breier U., Hensel G., Kumlehn J., Schubert I., Reiss B.
Stable gene replacement in barley by targeted double-strand break
induction. J. Exp. Bot. 2015;67(5):1433-1445. DOI 10.1093/jxb/
erv537.

Weising K., Schell J., Kahl G. Foreign genes in plants: transfer, structure,
expression, and applications. Annu. Rev. Genet. 1988;22:421-
477. DOI 10.1146/annurev.ge.22.120188.002225.

Woo J.W., Kim J., Kwon S., Corvalán C., Cho S.W., Kim H., Kim S.- G.,
Kim S.-T., Choe S., Kim J.-S. DNA-free genome editing in plants
with preassembled CRISPR-Cas9 ribonucleoproteins. Nat. Biotechnol.
2015;33:1162-1164. DOI 10.1038/nbt.3389.

Wu K.-M., Lu Y.-H., Feng H.-Q., Jiang Y.-Y., Zhao J.-Z. Suppression
of cotton bollworm in multiple crops in China in areas with
Bt toxin-
containing cotton. Science. 2008;321(5896):1676-1678.
DOI 10.1126/science.1160550

Zetsche B., Gootenberg J.S., Abudayyeh O.O., Slaymaker I.M., Makarova
K.S., Essletzbichler P., Volz S.E., Joung J., Oost J., Regev A.,
Koonin E.V., Zhang F. Cpf1 is a single RNA-guided endonuclease
of a class 2 CRISPR-Cas system. Cell. 2015;163(3):759-771. DOI
10.1016/j.cell.2015.09.038.

Zhang A., Liu Y., Wang F., Li T., Chen Z., Kong D., Bi J., Zhang F.,
Luo X., Wang J., Tang J., Yu X., Liu G., Luo L. Enhanced rice
salinity
tolerance via CRISPR/Cas9-targeted mutagenesis of the
OsRR22 gene. Mol. Breed. 2019;39:47. DOI 10.1007/s11032-019-
0954-y.

Zhang J., Zhang H., Botella J.R., Zhu J.-K. Generation of new glutinous
rice by CRISPR/Cas9-targeted mutagenesis of the Waxy gene
in elite rice varieties. J. Integr. Plant Biol. 2018;60(5):369-375. DOI
10.1111/jipb.12620.

Zhang P., Du H., Wang J., Pu Y., Yang C., Yan R., Yang H., Cheng H.,
Yu D. Multiplex CRISPR/Cas9-mediated metabolic engineering increases
soya bean isoflavone content and resistance to soya bean mosaic
virus. Plant Biotechnol. J. 2019;18(6):1384-1395. DOI 10.1111/
pbi.13302

Zhang Y., Bai Y., Wu G., Zou S., Chen Y., Gao C., Tang D. Simultaneous
modification of three homoeologs of TaEDR1 by genome editing
enhances powdery mildew resistance in wheat. Plant J. 2017;
91(4):714-724. DOI 10.1111/tpj.13599

Zhang Z., Ge X., Luo X., Wang P., Fan Q., Hu G., Xiao J., Li F., Wu J.
Simultaneous editing of two copies of Gh14-3-3d confers enhanced
transgene-clean plant defense against Verticillium dahliae in allotetraploid
upland cotton. Front. Plant Sci. 2018;9:842. DOI 10.3389/
fpls.2018.00842.

Zhang Z., Hua L., Gupta A., Tricoli D., Edwards K.J., Yang B., Li W.
Development of an Agrobacterium-delivered CRISPR/Cas9 system
for wheat genome editing. Plant Biotechnol. J. 2019;17(8):1623-
1635. DOI 10.1111/pbi.13088.

Zhou J., Peng Z., Long J., Sosso D., Liu B., Eom J.S., Huang S., Liu S.,
Cruz C.V., Frommer W.B., White F.F., Yang B. Gene targeting by
the TAL effector PthXo2 reveals cryptic resistance gene for bacterial
blight of rice. Plant J. 2015;82:632-643

Zhu T., Mettenburg K., Peterson D.J., Tagliani L., Baszczynski C.L.
Engineering herbicide-resistant maize using chimeric RNA/DNA
oligonucleotides. Nat. Biotechnol. 2000;18:555-558. DOI 10.1038/
75435.

Zhu T., Peterson D.J., Tagliani L., Clair G.S., Baszczynski C.L., Bowen
B. Targeted manipulation of maize genes in vivo using chimeric
RNA/DNA oligonucleotides. Proc. Natl. Acad. Sci. USA. 1999;
96(15):8768-8773. DOI 10.1073/pnas.96.15.87.

Zlobin N.E., Ternovoy V.V., Grebenkina N.A., Taranov V.V. Making
complex things simpler: modern tools to edit the plant genome.
Vavilovskii Zhurnal Genetiki i Selektsii = Vavilov Journal of Genetics
and Breeding. 2017;21(1):104-111. DOI 10.18699/VJ17.228.
(in Russian

Zong Y., Wang Y., Li C., Zhang R., Chen K., Ran Y., Qiu J.-L., Wang D.,
Gao C. Precise base editing in rice, wheat and maize with a Cas9-
cytidine deaminase fusion. Nat. Biotechnol. 2017;35(5):438-440.
DOI 10.1038/nbt.3811.

Zsögön A., Cermak T., Voytas D., Peres L.E. Genome editing as a tool
to achieve the crop ideotype and de novo domestication of wild
relatives: Case study in tomato. Plant Sci. 2017;256:120-130. DOI
10.1016/j.plantsci.2016.12.012

